# The complete chloroplast genome sequence of *Phalaenopsis wilsonii* Rolfe, a vulnerable wild moth orchid species (Orchidaceae)

**DOI:** 10.1080/23802359.2021.1923420

**Published:** 2021-09-13

**Authors:** Zhi-Feng Fan, Da-Yong Yu, Chang-Le Ma

**Affiliations:** a School of Landscape Architecture and Horticulture Sciences, Southwest Forestry University, Kunming, China; b Kunming University of Science and Technology, Kunming, China; c Southwest Landscape Architecture Engineering Research Center of National Forestry and Grassland Administration, Kunming, China

**Keywords:** *Phalaenopsis wilsonii*, chloroplast genome, breeding parent, phylogenetic analysis

## Abstract

*Phalaenopsis wilsonii* Rolfe is a vulnerable wild moth orchid species with important horticultural value. The complete chloroplast genome sequence of *P. wilsonii* was generated by de novo assembly using whole genome next-generation sequencing to provide genomic data for further conservation genetics, phylogeny and molecular breeding in *Phalaenopsis*. The complete plastome of *P. wilsonii* is 145,096 bp in length, containing two inverted repeats (IR) regions (24,787 bp), a large single-copy (LSC) region (84,688 bp), and a small single-copy (SSC) region (10,834 bp). The chloroplast genome encoded 119 unique genes, including 73 protein-coding genes, 38 tRNA genes, and 8 rRNA genes. The overall GC content of the whole genome is 36.9%. Phylogenetic analysis indicated *P. wilsonii* was closely related to *P. lowii*.

*Phalaenopsis* (Vandeae, Orchidaceae) is one of the most important and popular ornamental flowers in the world because of its beautiful appearance and high ornamental value. Based on morphology and DNA evidence, the *Phalaenopsis* genus is divided into four subgenera, namely *Parishianae*, *Phalaenopsis*, *Hygrochilus*, and *Ornithochilus* (Christenson 2001; Kocyan and Schuiteman 2014; Li et al. 2016). *Phalaenopsis wilsonii* Rolfe is belonging to subgenera *Parishianae*, which grow on trees or damp rocks under forests, mainly distributed among southwest China and northern India. *Phalaenopsis* is principally inhabiting the tropical regions, while *P. wilsonii* is also distributed in temperate zone. With purple pink flowers and abundant aerial roots, *P. wilsonii* is an important breeding parent of *Phalaenopsis* (Liu 2008). *Phalaenopsis wilsonii* has been categorized as vulnerable in China Species Red List (Wang and Xie 2004). Here, we report the complete chloroplast genome of *P. wilsonii*, which will provide genetic and genomic information for further conservation genetics, phylogenetic studies and future breeding in *Phalaenopsis*.

Fresh leaves of *P. wilsonii* were collected from Shangri-La county in northwestern Yunan province of China (99°29′31.41″E, 27°48′7.88″N, 2241 m). Voucher specimen (SWFU20200711MFY) was deposited in the Herbarium of Southwest Forestry University, China. Total genomic DNA was extracted from its fresh leaves using the Axygen® AxyPrep Multisource Genomic Miniprep DNA kit (Corning, Corning, NY) according to the manufacturer’s instruction. A pair-end (PE) library was constructed and sequenced using the Illumina HiSeq 2500-PE150 platform (Illumina, San Diego, CA). The clean reads was obtained from filtered raw reads using NGS QC Toolkit_v2.3.3 with default parameters (Patel and Jain 2012). The plastome was de novo assembled by NOVOPlasty (Dierckxsens et al. 2017), and annotated by Geneious Prime (Kearse et al. 2012) with the complete chloroplast genome sequence of *P. japonica* (NC_046808) as the reference. The complete chloroplast genome of *P. wilsonii* was submitted to GenBank with accession number MW194929.

The complete plastome of *P. wilsonii* is 145,096 bp in length, containing a large single-copy (LSC) region of 84,688 bp, a small single-copy (SSC) region of 10,834 bp, and a pair of inverted repeats (IR) regions of 24,787 bp. The overall GC content of the whole genome is 36.9%. In total, 119 unique genes were annotated, including 73 protein-coding genes (PCGs), 8 ribosomal RNA genes (rRNAs), and 38 transfer RNA genes (tRNAs). A total of 69 SSRs were discovered by the online software MISA-web (Beier et al. 2017). Among them, the numbers of mono-, di-, tri-, tetra- and penta-nucleotides SSRs are 49, 7, 4, 7, and 2, respectively.

To confirm the phylogenetic position of *P. wilsonii*, other 18 published complete chloroplast genomes from Orchidaceae were aligned by using MAFFT v.7 (Katoh and Standley 2013). *Tacca leontopetaloides* and *Burmannia disticha* were used as outgroups. A maximum-likelihood tree (Figure 1) was constructed with RAxML v8.2.11 (Stamatakis 2014) in which the GTR + G DNA substitution model was selected and all branch nodes were calculated under 1,000 bootstrap replicates. The phylogenetic tree showed that *P. wilsonii* was closely related to *P. lowii*. The result also appears in the ML tree constructed with ITS sequences (Tsai et al. 2003) and the combined plastid DNA (Tsai et al. 2010). The complete chloroplast genome sequence of *P. wilsonii* will provide useful information for further study on conservation genetics, phylogeny and molecular breeding in *Phalaenopsis* even in Orchidaceae.

**Figure 1.**
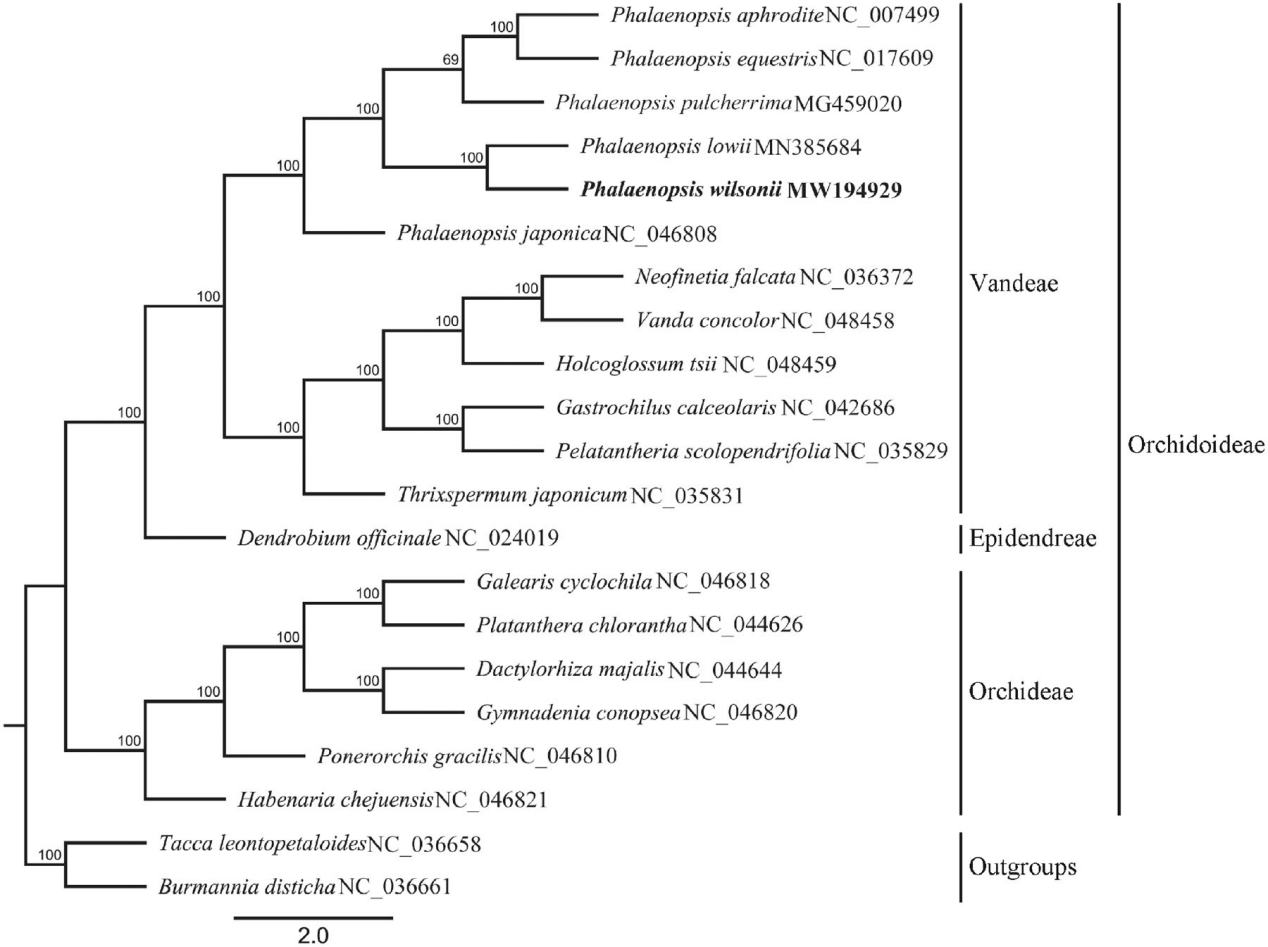
Maximum-likelihood phylogenetic tree reconstructed by RAxML based on complete chloroplast genome sequences from *P. wilsonii* and 18 other orchids, with *Tacca leontopetaloides* and *Burmannia disticha* as outgroups. Numbers on branches are bootstrap support values.

## Authors contributions

C. L. M conceived the study; D. Y. Y. collected the molecular materials; Z. F. F. drafted the manuscript; C. L. M. revised the manuscript. All authors provided comments and final approval.

## Data Availability

The data that support the findings of this study are openly available in GenBank at https://www.ncbi.nlm.nih.gov/, reference number (MW194929) (SRR12929239), or obtain from the corresponding author.
